# Sequential Laser–Mechanical Drilling of Thick Carbon Fibre Reinforced Polymer Composites (CFRP) for Industrial Applications

**DOI:** 10.3390/polym13132136

**Published:** 2021-06-29

**Authors:** Sharizal Ahmad Sobri, Robert Heinemann, David Whitehead

**Affiliations:** 1 Advanced Material Research Cluster, Faculty of Bioengineering and Technology, Jeli Campus, Universiti Malaysia Kelantan, Jeli 17600, Kelantan, Malaysia; 2 Department of Mechanical, Aerospace and Civil Engineering, The University of Manchester, Sackville Street Building, Sackville Street, Manchester M13 9PL, UK; david.whitehead@manchester.ac.uk; 3 Geopolymer and Green Technology, Center of Excellence (CEGeoGTech), Universiti Malaysia Perlis, Kangar 01000, Perlis, Malaysia

**Keywords:** carbon fibre reinforced polymer (CFRP), sequential drilling, delamination factor, thrust force and torque, hole depth, heat-affected zone (HAZ)

## Abstract

Carbon fibre reinforced polymer composites (CFRPs) can be costly to manufacture, but they are typically used anywhere a high strength-to-weight ratio and a high steadiness (rigidity) are needed in many industrial applications, particularly in aerospace. Drilling composites with a laser tends to be a feasible method since one of the composite phases is often in the form of a polymer, and polymers in general have a very high absorption coefficient for infrared radiation. The feasibility of sequential laser–mechanical drilling for a thick CFRP is discussed in this article. A 1 kW fibre laser was chosen as a pre-drilling instrument (or initial stage), and mechanical drilling was the final step. The sequential drilling method dropped the overall thrust and torque by an average of 61%, which greatly increased the productivity and reduced the mechanical stress on the cutting tool while also increasing the lifespan of the bit. The sequential drilling (i.e., laser 8 mm and mechanical 8 mm) for both drill bits (i.e., 2- and 3-flute uncoated tungsten carbide) and the laser pre-drilling techniques has demonstrated the highest delamination factor (*S_FDSR_*) ratios. A new laser–mechanical sequence drilling technique is thus established, assessed, and tested when thick CFRP composites are drilled.

## 1. Introduction

The Airbus A350 XWB is made of carbon fibre reinforced polymer composites (CFRP) (52% fuselage components and wing spars) [1], which overtake the Boeing 787 Dreamliner for the highest weight ratio of a CFRP aircraft, which was previously 50% [2]. This was one of the first commercial aircraft with composite wing spars. The Airbus A380 was one of the first commercial aircrafts to have a central CFRP wing box. It was also the first aircraft to have a smooth wing section rather than wings that are divided into sections span-wise. Drilling (which is one of the most common operations in manufacturing) creates holes for mechanical joints such as rivets, bolts, and screws [3]. Only the appropriate tool geometry, combined with the proper process conditions and ideal machining efficiency, can result in an acceptable level of damage [4].

The physical properties of fibre and matrix, fibre volume fraction, and fibre orientation are primarily determined by the properties of fibre reinforced polymer (FRP) materials. The machining of FRP composites is difficult and causes material damage in both mechanical and laser machining. The drilling of composites (particularly CFRP) is one of the most difficult processes to work with, and careful care must be taken to ensure protection from thermal shocks, fix problems with tools, avoid delamination and negate severe damage [3,4,5]. The optimum performance in the drilling of CFRP composites depends on the proper consideration of these conditions. Mechanical drilling requires the selection of optimum cutting operation parameters (i.e., cutting/spindle speed and feed rate) in order to avoid having any excessive forces affect the surface integrity of the CFRP composites [6]. Laser drilling depends on good thermal management or heat efficiency distribution towards the work piece, since the good thermal conductivity characteristics of CFRP make it prone to thermal damage at the surface (such as heat-affected zones (HAZ), porosity, etc.) [4,5,6]. The proper selection of parameters in laser drilling (such as laser power, scanning speed, gas pressure, and type) can be optimized in order to achieve an optimum performance [7].

Although researchers [8] have suggested some approaches to reduce the damage incurred to mechanical drilling (i.e., the use of support plates, special drill bits, and pre-drilled pilot holes), the optimization of cutting operating parameters (i.e., spindle speed and feed rate) is still considered to be the best approach to improve hole quality without the use of special equipment or tools. Some authors assume that optimising the cutting process parameters leads to better efficiency, since the use of a low feed rate and a high spindle speed favours minimal material damage and prolongs tool life [9,10].

Experimentally, laser power and cutting speed were found to be the most important parameters that influence surface roughness [11]. In addition, the most important parameters are kerf width, laser power, scanning speed, gas pressure, and duty cycle, while the taper angle has the same factors as the kerf width but with an additional consideration for pulse frequency [12,13,14]. The most important parameters for HAZ are laser power, scanning speed, gas pressure, pulse repetition, and duration of pulse. The discovery of the feasibility of the drilling process strategy has been explored extensively in the work of Sobri et al. [15], who have developed a new laser drilling strategy in their experiments. The spiral strategy was able to penetrate a 22 mm depth out of 25.4 mm thick CFRP in continuous wave mode, while only a 17 mm depth can be penetrated by the laser pulse mode.

According to Lauwers [16], the process of producing parts or work piece components by amalgamating different processes or machines during materials processing is typically called hybrid production/manufacturing. Based on the collective decisions of the International Academy for Production Engineering (CIRP): “Hybrid manufacturing processes are based on the simultaneous and controlled interaction of process mechanisms and/or energy sources/tools having a significant effect on the process performance”. Lauwers [16] classified hybrid processes into two groups. The first group is classified as the combination of two or more energy sources/tools that create a synergetic effect during the machining process. This group is narrowed down into two types: One type consists of “Assisted Hybrid Processes” such as laser-assisted turning/milling, vibration-assisted grinding, vibration-assisted EDM, and media-assisted cutting (high pressure jets, cryogenic cooling), which is also considered an assisted hybrid process wherein the amount of energy applied for the secondary process (the jet) is relatively high compared to that applied for a conventional process. The second type in this group consists of “Pure Hybrid Processes/Mixed Processes”. Examples of this type of process are the integration of grinding and spark erosion (which has grown to play an important role in the field), electrolysis (ECM) assisted wire–EDM, and electric and magnetic field-assisted finishing/polishing. The processes in the second group by Lauwers [16] are classified as process operations, wherein a controlled combination of effects occurs (normally performed in sequential operations). For example, the drilling of any material (the process of which involves laser drilling as pre-drill operation) that may start with a smaller diameter and then continue in a mechanical drilling operation with the desired diameter may be considered part of this group. This could be done vice versa depending on the outcome quality. These operations are conducted separately, during which it does not attach together during the machining process.

The phrase “simultaneous and controlled interaction” (extracted from the definition made by CIRP) indicates that the processes/energy sources should interact more or less in the same machining zone and at the same time [16]. This means that, if the implemented processes are conducted in a sequential way, they will not be considered as hybrid. A sequential method also has the potential to overcome the defects induced during the machining process. Okasha et al. [17] conducted research into the feasibility and the basic characteristics of a new approach for micro-drilling Inconel 718 alloy sheets at an acute angle, using a sequential laser and mechanical drilling. The process aimed to overcome the limitations of tip divergence and low tool stiffness in pure mechanical micro-drilling (especially for drilling at acute angles) and the issues of poor geometry, heat affected zones, recast layer formation, and back-wall damage that plague laser micro-drilling. The investigation focused on drilling at an inclined plane; a pilot hole was first drilled by a laser beam and then an end mill was used to machine the diffuser portion of the hole and provide a flat surface for the drill entrance side, and the holes were then finished via micro-mechanical drilling. The results of this sequential machining process were compared to those of mechanical drilling and laser drilling. The authors concluded that the complimentary process could be used to extend the lifespan of micro-drills and alleviate some of the size effect challenges and quality issues (i.e., burr size) that are driven by the rapid enlargement of the drill edge radius. It can also alleviate the thermal and geometric defects associated with laser drilled micro-holes.

The goals set for the development of a new method of machining are to enhance the level of surface integrity and to reduce waste. By combining laser technology with mechanical machining, both researchers and the industry are aiming at high quality, high productivity, and low cost, in comparison to other technologies (such as milling, shape-cutting, or water jet-cutting). Lauwers [16] stated that researchers have attempted to drill hardened steels and various ceramic materials using hybrid machining. Composites like a long-fibre reinforced aluminium matrix [18] and a particle reinforced aluminium matrix [19] were also investigated by applying hybrid machining. Other researchers investigated the hybrid or sequential machining of super-alloys [20,21], the hybrid machining of turbine airfoils [22], the sequential laser and EDM micro-drilling of fuel injection nozzles [23], and the most recent research attempt in sequential machining by Okasha et al. [17], as mentioned in the previous paragraph. These research attempts indicate that a tremendous reduction has been achieved in machining processing time, and production capacity has, therefore, significantly increased. Moreover, the most important benefits of hybrid or sequential machining include improved surface integrity (reduced surface roughness), the reduction in tool wear development (increased tool life performance), and the possible diminution in forces (reduced influence of thrust force and torque in drilling process).

This article explores the feasibility and basic characteristics of a novel sequential laser–mechanical drilling technique for drilling thick CFRP. The main machining process parameters must be chosen to ensure the avoidance of any major negative impact of the use of the sequential machining process, as each machining technology cuts holes of a different quality. In the future, these parameters (i.e., both laser and mechanical drilling in sequential or single machining processes) can be potentially improvised by other researchers in the research process of hybrid or sequential machining, as well as in a single machining process.

## 2. Materials and Methods

**Figure 1:**
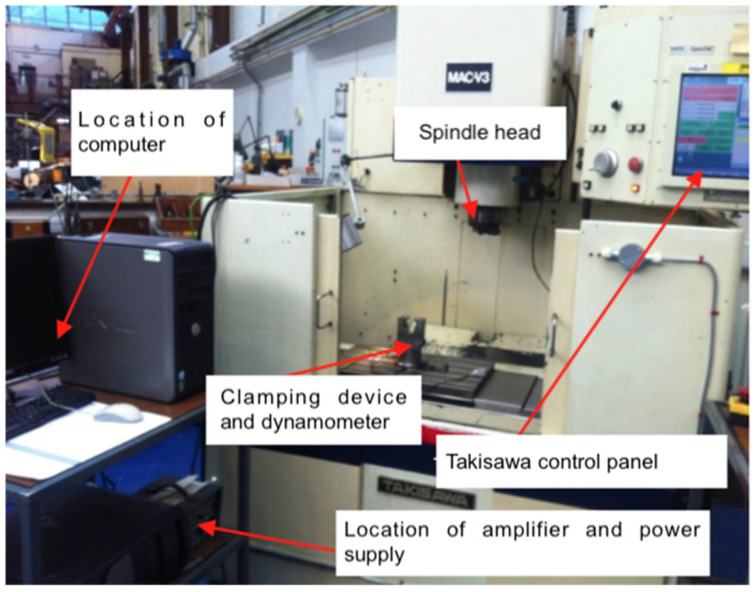
Takisawa MAC-V3 CNC machining centre.

**Figure 2:**
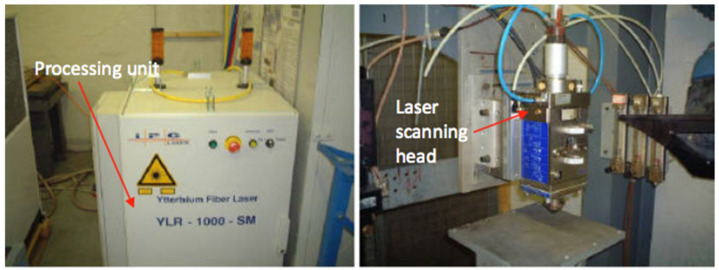
An IPG single-mode YLR-1000-SM 1 kW fibre laser [15].

Carbon fibre reinforced polymer composites (CFRP) provided by Airbus in Broughton, UK were used in the sequential drilling experiments. All machines and equipment were available and used at Department of Mechanical, Aerospace, and Civil Engineering, The University of Manchester. The technical specifications of CFRP are shown in Table 1 as follows:

All samples with an overall thickness of 25.4 mm CFRP were drilled using the Takisawa MAC-V3 CNC machining centre (Takisawa Machine Tool Co. Ltd., Okayama, Japan) for mechanical drilling (see Figure 1), and the IPG single-mode YLR-1000-SM (IPG Photonics (UK) Ltd., Bristol, UK) was used for laser drilling (see Figure 2). The maximum spindle speed and power of Takisawa MAC-V3 are 6000 rpm and 5.6 kW, respectively. IPG YLR-1000-SM was conducted in a continuous-wave (CW) fibre laser mode, and the technical specifications are as follows: single-mode emitting at near infrared; wavelength, λ = 1070 nm; laser power, P = 1 kW; and laser source = ytterbium doped. The focal length was 190 mm, and the focusing lens diameter was 38 mm. The focusing position can be changed coaxially (view window range: −20 to +10 mm). A Kistler dynamometer model 9271A (Kistler Instruments Ltd., Hook, UK) was used in these experiments, which it was fastened to the CNC machining table and connected with a Kistler multi-channel charge amplifier model 5001 (Kistler Instruments Ltd., Hook, UK) to record the thrust force and torque signals. The measuring time of the thrust forces and torques was 20 s while the sampling rate was taken at 1000 Hz. Various thrust force and torque values (with machining time) were plotted as waveforms. The average value of the maximum five peaks over a drilling cycle time in each wave diagram was used to investigate the influence of the cutting parameters on the drilling forces. This method is commonly used by a majority of researchers [5,24].

The quantification method for all hole-drilled surfaces was obtained by adopting the extension of the adjusted delamination factor (**S_FDSR_**) [25], and similar procedures for characterizing the damages were applied based on this reference. The extension of the adjusted delamination factor (**S_FDSR_**) method is able to measure the damage occurs inside the hole or at the cross-section area of the cylindrical hole, as shown in Equation (1). All samples were quantified at both holes (i.e., entry and exit), including the cross-section area, by using the Keyence Digital VHF-500X digital optical microscope (Keyence (UK) Ltd., Milton Keynes, UK).

(1) $$S_{FDSR}=\;\frac{D_{max}}{D_{0}}+\;\frac{A_{d}}{\left(A_{max}-\;A_{0}\right)}\;\left(F_{d}^{2}-\;F_{d}\right)+\;2\left(\frac{A_{dcs}}{\left[Length\;\left(l\right)\;x\;Width\;\left(w\right)\right]\;-\;\frac{2\pi rh}{2}}\right)$$

A quantitative approach based on the work of Li et al. [26] was applied for calculating the mechanical drilling energy (*E_m_*). Equation (2) shows the mechanical drilling energy:

(2) $$E_{m}\;=\;\int_{0}^{l}F\;dl\;+\;\int_{0}^{l}\frac{2\pi T}{f}\;dl$$

where *F* is the thrust force, *T* is the torque, *l* is the depth of drilling, and *f* is the feed per revolution.

Experimental studies used a combination laser and mechanical technique creating 8 and 10 mm holes in 25.4 mm thick CFRP. All parameters in Table 2 and Table 3 were identified based on the standard parameters obtained from previous research attempts [3,4,5,6,7,8,9,10,11,12,13,15,16,17,18,19,20,21,22,23] and modified to fit the current scenario based on machine and equipment capability. In this process, pilot holes were started with a 1 kW IPG fibre laser. The holes were then drilled with the Takisawa MAC-V3 CNC machining centre (Takisawa Machine Tool Co. Ltd., Okayama, Japan). Table 2 shows the parameters for the sequential laser–mechanical drilling process.

**Figure 3:**
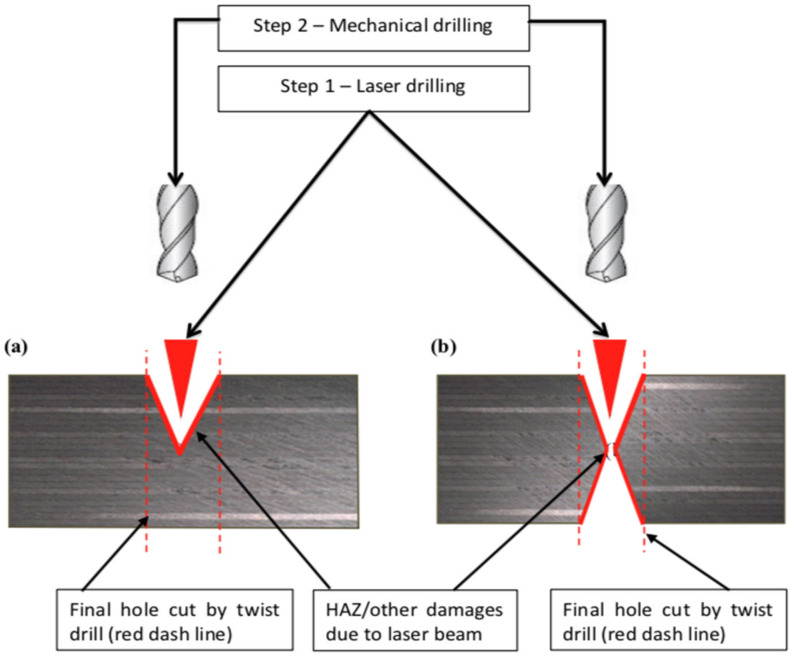
Illustration of sequential laser and mechanical drilling: (**a**) laser pre-drill on one side; and (**b**) laser pre-drill on both sides.

The spiral trepanning based on the work of Sobri et al. [15] was adopted as a drilling movement for the laser drilling process due to the successful penetration of a hole more than 20 mm in depth. The first stage was to perform a hole quality assessment wherein a laser was used as the initial step, followed by using an 8 mm-diameter mechanical drill to complete the hole (i.e., in the final step). The next stage produced a hole 10 mm in diameter, while the final step produced a hole 8 mm in diameter. The third stage was initiated by a 6 mm diameter laser drilling, which led to a final hole diameter of 8 mm. These settings were intended to demonstrate whether: (a) an 8 mm laser pre-drilled hole can be cleaned off with an 8 mm drill; (b) how much bigger than 8 mm one would have to drill to eliminate any damage introduced by an 8 mm laser drilled hole; and (c) whether to create an 8 mm final hole, the laser pre-drilled hole might have to be smaller. When pre-drilling a laser-drilled hole with a 6 mm diameter, the reason for this is to optimise the drilling quality by minimising the HAZ or other damage and then to assess the effects of the drilling forces during the mechanical drilling process. All holes were created in two separate approaches: the first approach was conducted by drilling from one side (i.e., at the top only), while the second approach was conducted by drilling from both sides (i.e., top and bottom). As can be seen in Table 3, “single-side” and “double-side” are the parameters for the first and second approach, respectively. Figure 3 shows an illustration of the sequential machining process. The aim of using three different sequence drilling arrangements (i.e., Laser–Mechanical: 8–8 mm, 8–10 mm, 6–8 mm) was to identify which one was more feasible in reducing the damage done by the laser (i.e., HAZ, fibre uncut, etc.) as well as by the cutting forces. A number of flutes (i.e., 2- and 3-flute) were also investigated in both approaches to examine the efficiency of the cutting edge on the consistency of the hole at the entrance and exit sides. The 2-flute uncoated tungsten carbide (WC) has a helix angle of 35°, a point angle of 118°, a drill length of 62mm (diameter = 8 mm) and 71 mm (diameter = 10 mm), and a chip flute length of 75 mm (diameter = 8 mm) and 87 mm (diameter = 10 mm). For the 3-flute WC drill bit geometry, the angle of the helix is 43°, the angle of the point is 150°, the length of the drill is 35 mm (diameter = 8 mm) and 39 mm (diameter = 10 mm), and the length of the chip flute is 48 mm (diameter = 8 mm) and 55 mm (diameter = 10 mm).

The two-step drilling process that combines laser and mechanical drilling was possible to achieve, but the big challenge was the accuracy of re-positioning the work piece. The first challenge was in drilling “double sided” holes because the work piece needed to be turned over manually and then positioned such that the laser was aligned with the already drilled blind hole. The second challenge occurred during the subsequent drilling process when aligning the mechanical drill to the pre-drilled hole. Drilling from one side by a laser always resulted in a blind hole. During the laser pre-drill step, the work piece was clamped and put on the CNC machining table. A laser guide (i.e., pointer) was used to manually align both holes to indicate how accurate the position was. In this case, the inaccuracy was found to be 0.5 mm. For the next step, the fully drilled hole was aligned manually in a Takisawa CNC machining centre to drill the final hole using the twist drill.

**Figure 4:**
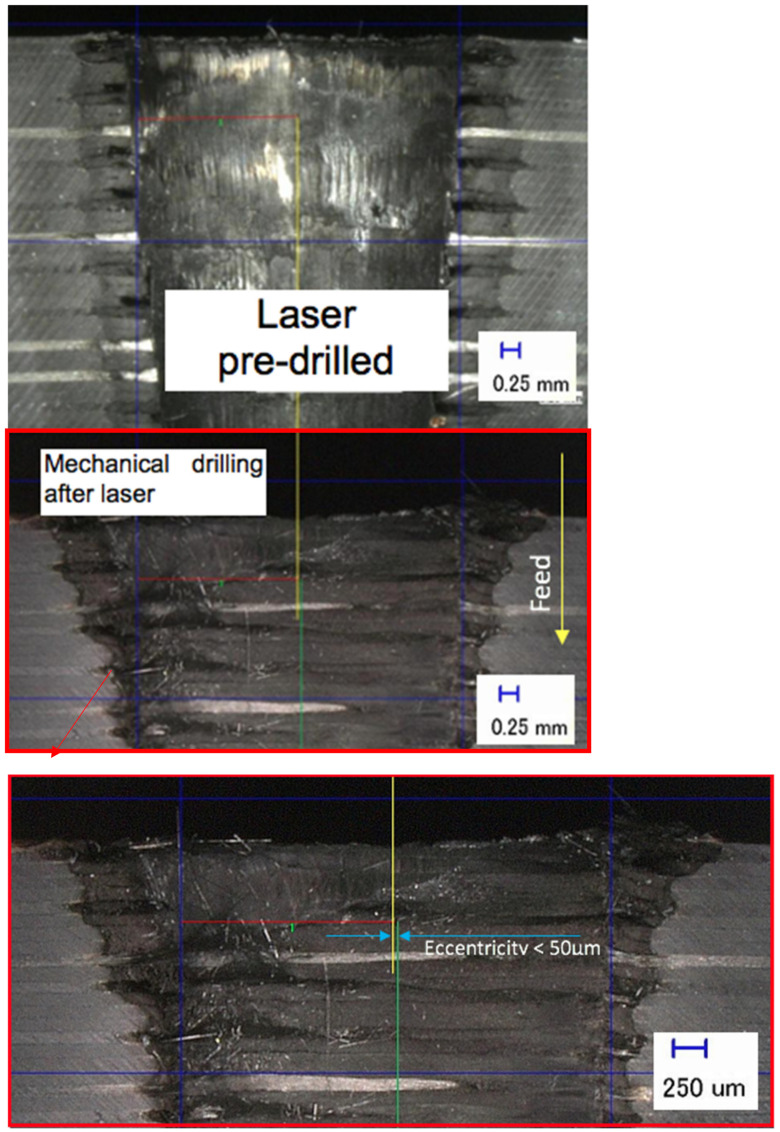
Example of sequential drilling alignment accuracy measurement.

The laser holes drilled from both sides must be symmetrical, which, in extreme cases, was ensured in the subsequent drilling step to ensure that the centres of the two holes were precisely matched and collected accurate data on the drilling forces. The work piece was rotated manually from the bottom position to the top position, which was done by stopping the laser machine for 50 s and fixing on the laser CNC machining centre again for the next laser-drilling step. In order to ensure a precise alignment of the centre of the two holes, a further inspection was carried out by cutting the work piece in a cross-section and measuring the eccentricity by visually determining the side walls and assuming that the axis lies precisely in the middle between the two holes. This was used to prove that the manual flipping of the work piece was accurate to approximately 50 microns. Figure 4 shows an example of measuring the eccentricity between the two holes, and was recorded between 15 and 40 μm during the inspection. The first step was to determine the diameter of the hole at the top by finding two points of the hole edges in order to locate the middle of the hole, as seen in Figure 3 (i.e., laser pre-drilled). Next, the diameter was measured at the bottom (i.e., within the mechanical drilling after laser). Finally, after obtaining the centre of the hole on each side, the eccentricity was determined. This is important for recording cutting forces by means of mechanical drilling in order to avoid the presence of unnecessary materials on one side and to ensure the geometrical accuracy of both holes.

## 3. Results and Discussion

**Figure 5:**
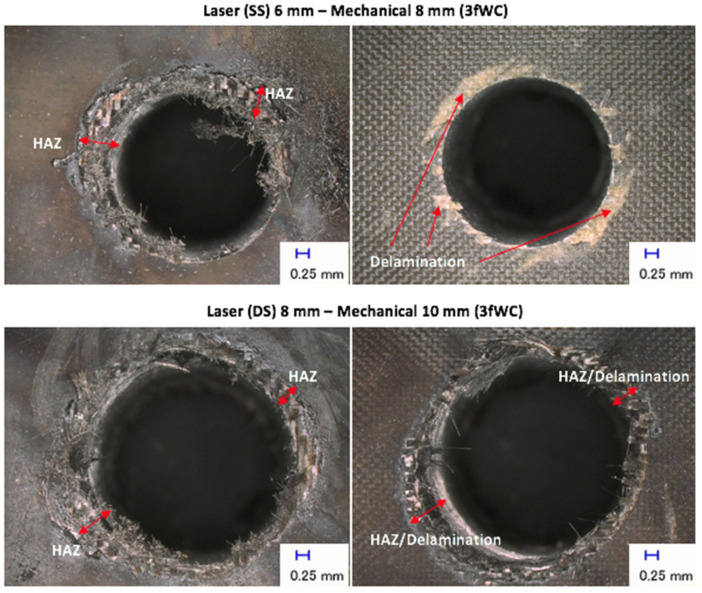
Typical examples of sequential laser–mechanical drilling results.

**Figure 6:**
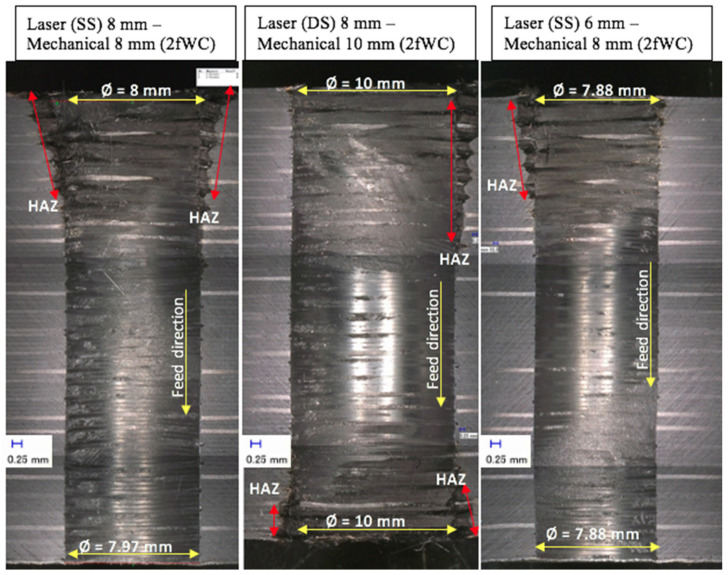
Typical cross-section views of sequential laser–mechanical drilling results.

Figure 5 shows examples of damages at the hole entry and exit points, with various three sequential arrangements. The picture on the left is of the entry side while that on the right is of the exit side. These pictures show the typical damages that occurred for both entries in all sequential drilling arrangements, and damages at both entries yielded higher values for quantifying the **S_FDSR_** ratio (later as shown in Figure 9). The highest **S_FDSR_**, as well as the unattended HAZ, was expressed by the sequential laser 8 mm—mechanical 8 mm for both tools and laser pre-drilled strategies (i.e., single-side (SS) and double-side (DS)). This observation is also present in the experimental work of Sobri et al. [6]. This result could be due to the presence of the pre-drilled holes, because the drill bit tool diameter has the same diameter and therefore is unable to eliminate the HAZ contributed by the laser beam. The tools with a 10 mm diameter used for drilling 8 mm pre-drilled holes (including an 8 mm drill bit used to drill 6 mm pre-drilled holes) managed to reduce the amount of HAZ left after laser pre-drilling. However, there was a small amount of HAZ still left on the hole’s periphery. This was not significant as experienced by the sequential laser 8 mm—mechanical 8 mm. The quantification of HAZ was included the measurement of the HAZ area inside the hole. Figure 6 shows the typical results of sequential drilling in various arrangements when the work piece samples were cut off cross-sectionally. Each micrograph provides the hole diameter (i.e., Ø in mm) and an indication of the HAZ area, including the feed direction from top to bottom. The HAZ inside the hole was reduced significantly by an overall percentage of 62.5% compared to the HAZ at the hole entry and exit, which was reduced by 48.7% after mechanical drilling. Based on this figure, double-sided laser pre-drilled holes experienced the worst HAZ occurrence after mechanical drilling took place, a result that is also corroborated with the **S_FDSR_** results. The HAZ created is wider than the overlap between the laser-pre-drilled hole and the twist drill. This is because the second laser drilling process created a HAZ much wider than that created by the first process (i.e., SS drilling gives a smaller HAZ than DS drilling) and the HAZ after drilling is greater for DS than for SS. In other words, an even larger drill diameter is needed to get rid of the HAZ (created by DS laser pre-drilling). Moreover, by comparing between the three arrangements, the sequential laser 6 mm—mechanical 8 mm for both drill bits (as well as the laser pre-drilled strategies (i.e., SS and DS)) was found to be the most favourable selection for a better hole. Damages were also discovered when observing the hole entry and exit as well as inside the hole. The most typical damages occurred in cases (such as HAZ existence after mechanical drilling, delamination being seen at hole exit with the approach of the single-side laser pre-drilled strategy, and a few fibres remaining uncut). All of these were experienced in a similar manner to the phase 1 experiments, excluding the existence of HAZ.

**Figure 7:**
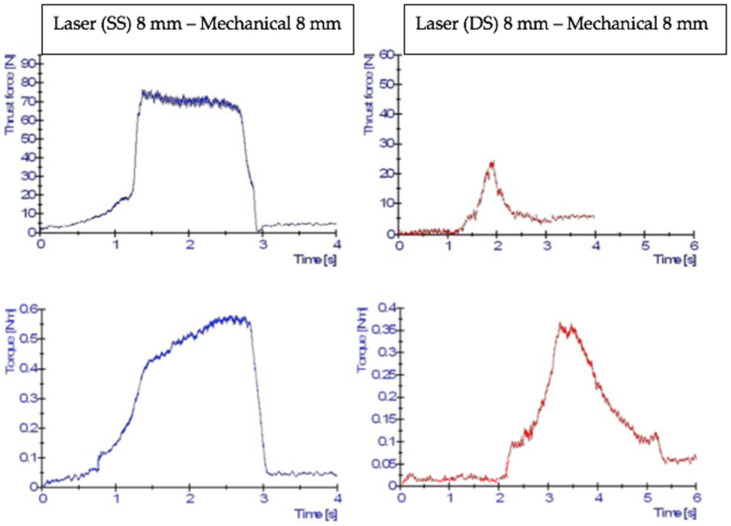
Typical examples of thrust force and torque signal diagrams for uncoated tungsten carbide (WC).

Figure 7 shows typical examples of the thrust force and torque signals. The blue signal in each diagram indicates the result of the drilling force after the laser drilling process was conducted using the single-side (SS) approach, whereas the red signal shows the double-side (DS) approach for laser drilling. As shown in the figure, the single-side approach exhibited a slightly longer interaction time between the tool and work piece compared to the double-side approach due to the material remaining in the hole. The single-sided holes confronted the drill with a more “traditional looking” or similar force curve [8,9,10], generating a force curve that looks more like the traditional curve. At the beginning of drilling, the chisel edge was penetrating the work piece’s layers when it reached the middle of the hole, which caused the thrust force to rise quickly. The torque rose slowly because of the smaller cutting forces present at the chisel edge and the proximity of these forces to the centre of the drill. The torque started to increase rapidly as the cutting edges engaged in the centre of the hole (i.e., the first point to cut the layer). The only difference between the single-sided and double-sided approaches was found at the region where the drill bit was fully engaged in cutting the layers, wherein the double-sided approach left a few layers (i.e., after laser pre-drilling) at the centre of the hole. The double-sided approach showed a kind of double taper (or blind form) entry and exit holes, which caused the drilling to progressively engage until it reached the centre. After that, it progressively disengaged as the drill went further down the hole. The force curve was shorter in this region compared to the single-side’s force curve and was similar to single-side in that the force values were lower compared to the single-side. Hence, there was a gentle rise and a gentle fall. During the drilling process, the tool absorbs approximately 50% of the mechanical energy provided for CFRP composites [5,9] and the remainder is converted into heat, which is then transferred and distributed equally to the chip and work piece [5,9,18]. Since thrust force and torque are produced by the cutting action of the two or three primary cutting edges, it is believed that 50% is divided between three cutting edges (i.e., 16.7% heat generated for each cutting edge), while for 2-flute uncoated WC, each cutting edge contributes 25% heat generated. It is possible that the heat produced in each cutting edge of a 2-flute uncoated WC produces additional stresses between the tool and the work piece. To clarify this, the mechanical drilling energy (E_m_) equation developed by Li et al. [26] was used. The mechanical drilling energy (E_m_) was 81 J for 2-flute uncoated WC and 99 J for 3-flute uncoated WC, respectively. The E_m_ was calculated to be 71.57 J, 75.31 J, and 76.23 J for 2-flute uncoated WC in three separate arrangements with single-side laser pre-drilled holes (i.e., SS laser 8 mm–mechanical 8 mm, SS laser 8 mm–mechanical 10 mm, and SS laser 6 mm–mechanical 8 mm, respectively). The E_m_ values for double-sided laser pre-drilled holes were 60.64 J, 69.3 J and 60.92 J, respectively. The mechanical energy drilling (E_m_) values for 3-flute uncoated WC with single-side laser pre-drilled holes were found to be 63.77 J, 73.84 J, and 56.19 J, respectively, while the E_m_ values for double-side laser pre-drilled holes were 32.58 J, 34.8 J, and 44.34 J, respectively.

**Figure 8:**
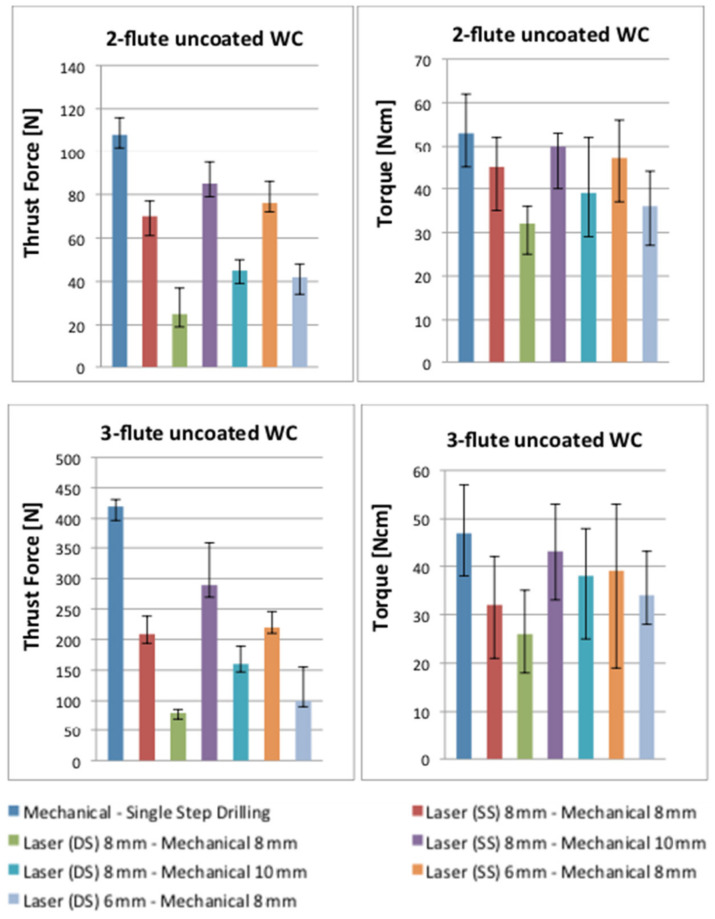
Thrust force and torque results from sequential drilling.

Figure 8 shows the comparison of thrust force and torque results between purely mechanical drilling and sequential drilling for 2- and 3-flute uncoated WC, the values of which were extracted from the force signal curves. As shown in this figure, the blue bar shows the result of a 100% mechanical drilling process in a single step strategy while the various coloured bars illustrate the sequential laser–mechanical drilling results when the laser drilled the work piece for single-(SS) and double-sided (DS) approaches, respectively. Based on the results in this figure, the sequential laser–mechanical drilling process decreases the thrust force and torque compared to the purely mechanical drilling process. These changes occurred due to the already existing hole allowing the chisel edge to penetrate, with no contact, towards the work piece. Thus, the thrust force was reduced. However, the cutting edges still had to remove material around the circumference. In the case, the laser hole was smaller than the drill, and the drill had to remove more material as compared to an 8 mm laser hole and an 8 mm twist drill. Another finding found in the experiment was that the reduction in cutting forces also relied on the pre-drilled hole diameter. Based on the figure, the pre-drilled hole diameter of 6 mm reduced cutting forces more than did a hole diameter of 8 mm for both drill bits. It could be possible that the thrust force is a uniformly distributed load over the drill bit diameter instead of a concentrated load (i.e., the thrust force in the drilling operation comes through the centre of the drill bit) and this theory can be supported by various researches [5,8,9,10]. Furthermore, this result indicated that a higher diameter leads to a higher thrust force spread over the drill bit diameter. With the assistance of pre-drilled holes, the thrust force was reduced due to a small amount of material being removed earlier. The 3-flute uncoated WC achieved better results than did the 2-flute, which significantly decreased the cutting forces. In this scenario, the 3-fluted drill was used with a lower feed rate per edge, which could have resulted in this tool generating lower cutting forces (i.e., torque, in particular).

**Figure 9:**
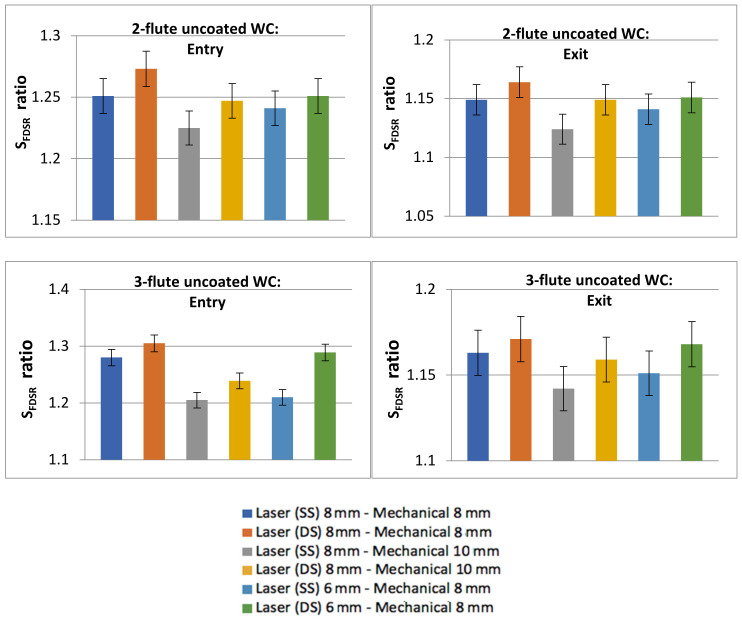
The *S_FDSR_* ratio results from sequential drilling.

**Figure 10:**
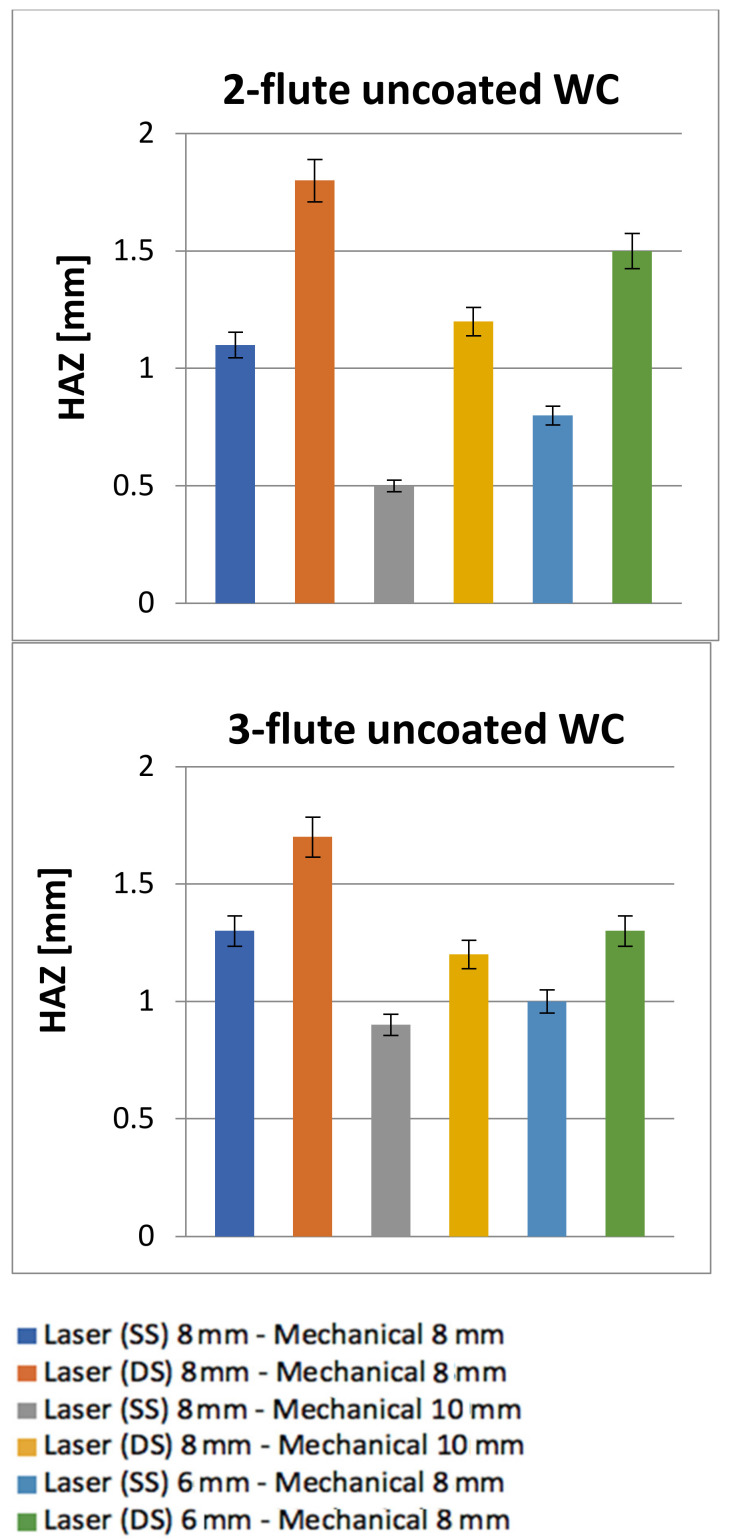
The results of the remaining heat-affected zones (HAZ) left after mechanical drilling.

Figure 9 shows the results of the *S_FDSR_* ratio. The top diagrams are the results for the 2-flute uncoated WC while the bottom diagrams are for the 3-flute uncoated WC results. Six sequential arrangements are highlighted in each diagram. A similar category in the work of Sobri et al. [16] was also applied in these experiments for the requisite level of delamination. Based on the results, the entry side for both tools experienced severe damage, which it is categorised under the third level of delamination (i.e., a ratio ≥1.201) and is highly unfavourable as a good hole. The exit side results were achieved in the second level of delamination (i.e., a range between a ratio of 1.101 and a ratio of 1.200), which was considered to be a good hole and rectification on the hole was deemed not essential. The entry side exhibited a higher *S_FDSR_* than did the exit side, which could have been caused due the existence of the protection layer coated on the first layer. The protection layer is still unclear as to which type of surface coating material has been used, as this has never been disclosed by Airbus. There are variations in the surface layers applied by the industry, and their properties can influence the performance of the hole and the consistency of the edges [4,5,10]. This surface layer can cause problems such as splintering, but it may help to minimise delamination. Another explanation for this result is the formation of HAZ created by the laser (which contributes more damage than does mechanical drilling) and the fact that the interaction effect between the laser and the surface properties leads to a significantly damaged region. Furthermore, holes with double-side laser pre-drilling experienced the worst results compared to other holes with single-side laser pre-drilling. HAZ remains on the hole periphery and Figure 10 show the average HAZ duration values, of which all are quantified (i.e., based on the size of gap from the circumference border of the drilled holes to the final point of HAZ). Other holes were also left with HAZ, which was also not completely removed by the twist drill during the mechanical drilling process. HAZ occurred dominantly at the entrance and exit holes, whereas HAZ created by laser inside the hole was significantly reduced by mechanical drilling, which was capable of removing HAZ, especially in the middle of the hole, as can be seen in Figure 6.

**Figure 11:**
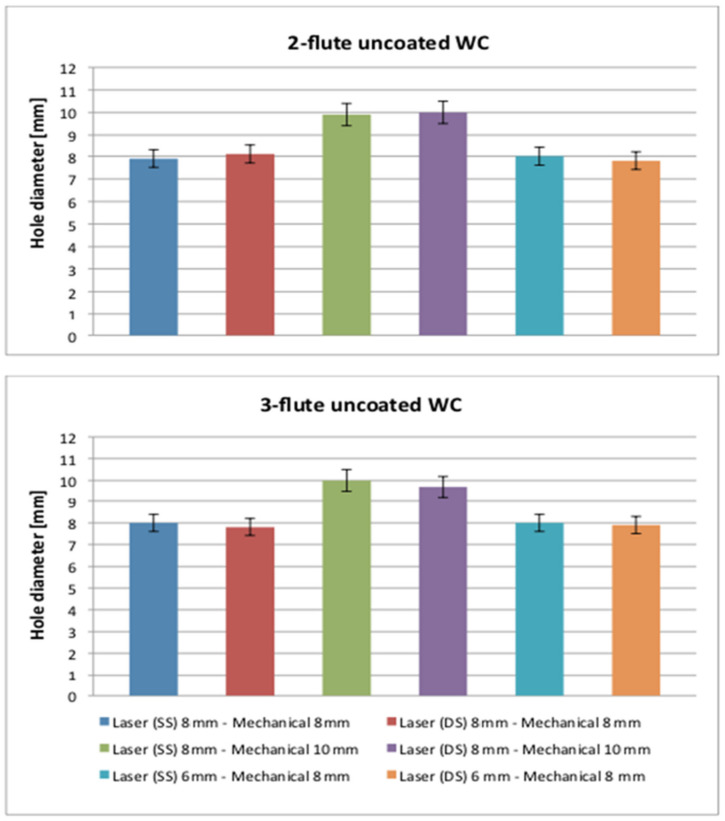
The results of the hole diameters resulting from sequential drilling.

Furthermore, this outcome can be seen clearly from the results of the laser (SS and DS) 6/8 mm–mechanical 8/10 mm arrangements because it can produce better results compared to the laser (SS and DS) 8 mm–mechanical 8 mm arrangement. Spiral trepanning was capable of cutting different diameter sizes, and this indicated that the larger the diameter required, the wider the HAZ would be. This result also occurred in the work of Sobri et al. [11]. In addition, double-side laser pre-drilled holes were still at the top of the chart. The HAZ adjacent to the hole circumference for both entries was a critical issue for sequential drilling with the application of double-side laser pre-drilling because the protection layer at the initial and the final layer could have a significant effect on each hole. Apart from these findings, the diameter of the hole was also measured, and the final diameter range reported was between 7.88 mm and 8.04 mm for the 8 mm diameter tool and the range for the 10 mm diameter tool was between 9.90 mm and 10 mm, as shown in Figure 11. This indicates that there was no critical issue with respect to the taper problem on each hole and that most of the holes were created at a nominal diameter size (i.e., 8 mm and 10 mm). Despite this, the percentage of error in diameter (i.e., the difference between the required/nominal diameter and the actual diameter of the hole produced) was extremely small, contributing 1.5% (i.e., the highest value for the undersized hole) and 0.5% (i.e., the highest value for the oversized hole) to the final 8 mm diameter, while 1% (i.e., the highest value for the undersized hole) was contributed to the 10 mm diameter. For some of the applications envisaged, this was perceived to be within the appropriate boundaries of tolerance.

A possible way to explain these results is that the laser pre-drilling softened the matrix excessively, which prevented the matrix from properly transferring the load to the fibres. The uncut fibres became more susceptive to deformation due to a decrease in the work piece’s thickness (i.e., layers) during the second step of sequential drilling (i.e., the mechanical drilling process). Moreover, the damages were due to the nature of the drilling operation; the rotating cutting edges were constantly changing their relative positions with respect to reinforcement and advancing through the thickness helically. It is worth noting that the size of a laser pre-drilled hole is ideally reduced in order to eliminate damages (i.e., HAZ and delamination). For example, if the final diameter is 10 mm, it is advisable to reduce the laser pre-drilled hole size from 8 mm to 6 mm, which leaves 4 mm to be cut by mechanical drilling to make sure that the damages are completely removed. The subsequent drill diameter needs to be large enough to remove all of the HAZ so that the final hole is freed from HAZ. Consequently, if a given final hole size of “X”, a laser-drilled hole needs to be produced that is accordingly much smaller. In the current case, it is assumed that by applying the 6 mm laser setting in combination with a 10 mm drill. a good quality hole will likely be achieved. Apart from the reduction in the laser pre-drilled hole diameter, it could be possible that the fibre layers in the first and final ply are in a uni-directional-ply form and might reduce the damages and the cutting forces by using the peel–ply at both layers without any coating material for protection, which may potentially reduce the damages as well.

**Figure 12:**
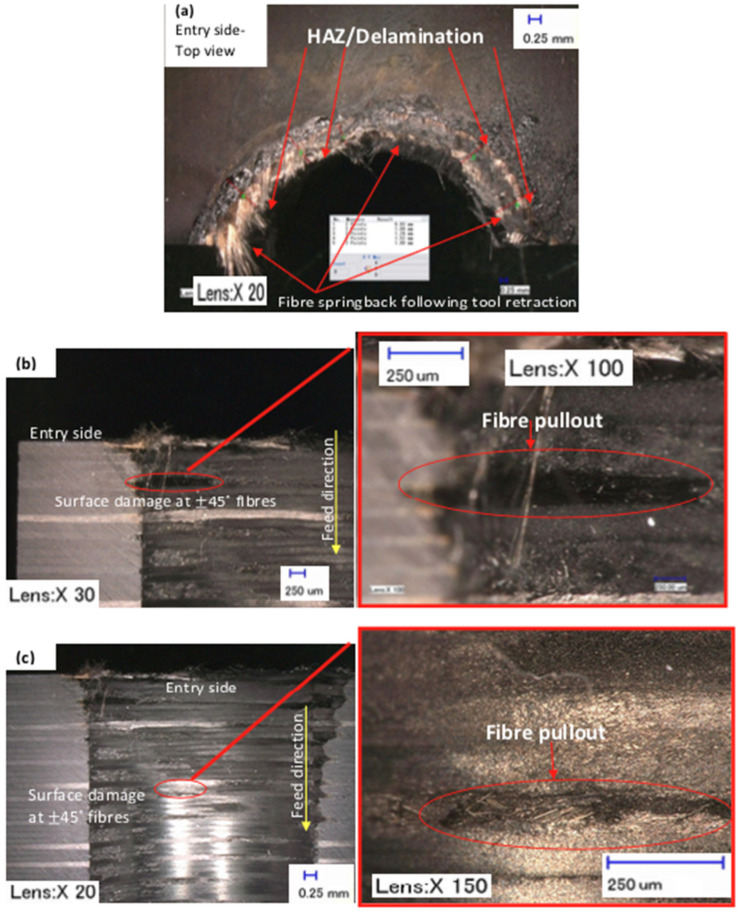
Typical examples of damages occurred for sequential laser (DS) 8 mm–Mechanical (3fWC) 10 mm: (**a**) top view after cross-section cut; (**b**) fibre pullout at the edge; and (**c**) fibre pullout inside the hole.

When the softening of the resin became excessive due to the heat generated by the laser beam, the fibre spring back was significantly apparent after the second step of sequential drilling, as can be seen in Figure 12 and Figure 5. Figure 12a shows the HAZ, as well as the possibility that delamination and fibre spring back occurred for sequential drilling, which was adversely affected since some of the fibres were deflected during cutting and sprung back when the tool was retracted. Figure 12b shows a typical example of the hole edge quality contributing to machining induced surface debris due to an inefficient matrix-fibre load transfer, together with the characteristic failure behaviour of fibre orientation. The region with a ±45° fibre orientation at ply number 5 (i.e., see Figure 12b) and the same fibre orientation at ply number 21 (i.e., see Figure 12c) developed extensive fibre pullout, which created crater-type surface defects. In this fibre orientation, the fibres that fractured similarly occurred at different ply numbers according to their length and, hence, produced irregular surfaces.

An implication of these findings is that the sequential drilling concept successfully reduced the cutting forces by an overall average of 61% based on the experimental data of the drilling experiments. Therefore, it can be foreseen that the reduction in cutting forces in the application of sequential drilling would tend to result in a comparable reduction in the generated heat, decreasing the thermal stresses in the twist drill bit. The reduction of thermal stresses is capable of distorting activated wear mechanisms, and, in turn, would potentially enhance the tool’s life. The main limitation of this concept of sequential drilling is the time required for rotating the work piece from the bottom position to the top position, which took at least 50 s to ensure that the work piece was correctly in position. Apart from this analysis, tool life evaluation (as well as flank wear analysis) was not included in these experiments due to a limited number of tools per task (i.e., one tool drilled two holes, which hardly showed flank wear development) and materials. Future studies can be conducted by including the tool wear evaluation and would be of interest.

## 4. Conclusions

A novel sequential laser–mechanical drilling method had been developed, evaluated, and tested in the drilling of 25.4 mm thick CFRP composites. At present, this is the first attempt ever made to combine both machining methods and the first ever reported on the sequential machining of thick CFRP composites. The sequential drilling method successfully reduced the thrust force and torque for mechanical drilling by an overall average of 61%, resulting in high productivity, decreasing the thermal and mechanical stresses in the cutting tool, and in turn, promoting a higher tool life. For future studies, the damages (i.e., HAZ and delamination) may potentially be removed by reducing the size of the laser pre-drilled hole in order to leave 4 mm of materials to be cut via mechanical drilling (i.e., if the final diameter is 10 mm, it is recommended to drill a 6 mm diameter hole using the laser, followed by mechanical drilling with the 10 mm diameter drill bit for final diameter). In addition to that, it is interesting to study the potential of drilling thick CFRP composites by having the samples with a unidirectional-ply form at the first and final layer or the application of peel–ply at both layers without a coating material, which may also potentially reduce the cutting forces as well as the damages, especially at the hole entry and exit.

## Figures and Tables

**Table 1:** The material.

**Material**	Carbon fibre reinforced polymer (CFRP)
**Grade**	M21
**Yield Strength**	835 MPa
**Density**	2.06 g/cm^3^
**Lamina Orientation Arrangement**	0°/90°/−45°/90°/45°/90°/−45°/90°/45°/90°/−45°/90°/0°
**Thickness**	1 ply = 0.22 mm

**Table 2:** The process parameters for sequential drilling: (a) pre-drill step–laser drilling; and (b) final step–mechanical drilling.

(a)	
Parameter	Parameter Input Value/Setting
Laser Power	900 W
Scanning Speed	10 mm/s
Type of Assist Gas	Argon
Gas Pressure	8 bar
Nozzle Diameter	1 mm
Stand-Off Distance	1 mm
Focal Plane Position (FPP)	−12 mm
Focal Length	7.5″
Focal Lens Diameter	1.5″
Beam Spot Diameter	70 μm (at reference point, FPP = −12 mm)
**(b)**	
**Parameter**	**Parameter Input Value/Setting**
Cutting Speed	140 m/min or 5570 rpm
Feed Rate	0.096 mm/rev
Tool Type	Uncoated tungsten carbide (WC)
Diameter	8 and 10 mm
Cutting Condition	Dry

**Table 3:** The sequential drilling arrangement.

		No. of Flute	
		2-flute (2f)	3-flute (3f)
**Sequential** **arrangement**	(1) Laser–8 mm (2) Mechanical–8 mm	single-side double-side	single-side double-side
	(1) Laser–8 mm (2) Mechanical–10 mm	single-side double-side	single-side double-side
	(1) Laser–6 mm (2) Mechanical–8 mm	single-side double-side	single-side double-side

## Data Availability

The data presented in this study are available on request from the corresponding author.
